# The crisis is over, long live the crisis: mental health in emerging adulthood during the course of the COVID-19 pandemic

**DOI:** 10.3389/fpsyg.2024.1283919

**Published:** 2024-01-31

**Authors:** Janine Wirkner, Eva-Lotta Brakemeier

**Affiliations:** Department for Clinical Psychology and Psychotherapy, Institute for Psychology, University of Greifswald, Greifswald, Germany

**Keywords:** COVID-19 pandemic, mental health, emerging adulthood, depression, anxiety

## Abstract

**Introduction:**

As a multidimensional stressor, the COVID-19 pandemic posed a significant threat to mental health, with studies showing younger age groups to be particularly vulnerable. Thus, this study aimed to monitor mental health, potential risk/protective factors, and pandemic-related variables among young university students during the pandemic.

**Methods:**

Students of the University of Greifswald (M age = 23.0 years, 73.9% female) participated in five cross-sectional online surveys in December 2020 (*N* = 1,127), March 2021 (*N* = 760), June/July 2021 (*N* = 531), December 2021 (*N* = 1,226), and December 2022 (*N* = 814). Sociodemographic data, depression and anxiety severity, loneliness, quality of life, coping strategies, resilience, self-esteem, and emotion regulation were measured. First, results from December 2020 were compared to pre-pandemic normative data. Second, the time course during the pandemic was analyzed. Third, linear models were calculated to examine the influence of risk and protective factures on depression and anxiety severity.

**Results:**

Higher levels of depression, anxiety, and loneliness, as well as lower levels of self-esteem, physical and mental health, social relationships and well-being were found in December 2020 compared to pre-pandemic. Levels of depression and anxiety severity peaked in December 2022. Female sex, loneliness, and previous mental treatment showed associations with higher depression and anxiety severity, while higher self-esteem, resilience and use of reappraisal strategies appeared to act as protective factors.

**Discussion:**

The study indicates the pandemic’s detrimental impact on students’ mental health and quality of life. Identified risk and protective factors provide guidance for tailored prevention and treatment, as well as the design of measures for future pandemics and other crisis.

## 1 Introduction

Following its outbreak, the COVID-19 pandemic has been termed as a multidimensional stressor, hypothesizing that apart from its impact on physical health, the pandemic also poses a serious threat to mental health (Brakemeier et al., 2020; Gruber et al., 2020). This hypothesis is based on studies of previous epidemics (SARS-CoV-1 in 2003 and MERS-CoV in 2012; Lee et al., 2007; Esterwood and Saeed, 2020; Vindegaard and Benros, 2020) and the financial crisis between 2007 and 2009 (Katikireddi et al., 2012; Boyce et al., 2018; Forbes and Krueger, 2019). In particular, the global spread of the virus, the long-term restriction measures (including lockdowns), and the unpredictable duration made the pandemic appear to be a multidimensional stressor (Brakemeier et al., 2020; Gruber et al., 2020; Holmes et al., 2020).

However, the expected “tsunami” of mental illness did not hit the general population (Shevlin et al., 2021). By comparing mental health before the pandemic and during the first year of the pandemic, some studies found an increase in psychological distress (McGinty et al., 2020; Pierce et al., 2020; Niedzwiedz et al., 2021; Ramiz et al., 2021), but others found no changes in depression symptoms (Castellini et al., 2021) or even a decrease in depression symptoms, or comparable or lower anxiety levels (van der Velden et al., 2020; Hyland et al., 2021). There were also conflicting results regarding the subsequent course of the pandemic (Wirkner et al., 2022), with continuously elevated scores for psychological distress (McGinty et al., 2022), no changes in depression and generalized anxiety disorder (Hyland et al., 2021), or even a decrease in generalized anxiety and depression (Fancourt et al., 2021; Varga et al., 2021). In addition to varying survey methods and study samples (Pierce et al., 2020), fluctuating COVID-19 incidence and severity, as well as specific risk and protective factors, may explain this heterogeneity in results (Shevlin et al., 2021; Wirkner et al., 2022).

Regarding risk factors, there is increasing evidence that young age (Bu et al., 2020; Pierce et al., 2020; Fancourt et al., 2021; Varga et al., 2021), female sex (Pierce et al., 2020; Niedzwiedz et al., 2021; Ramiz et al., 2021; Bendau et al., 2021b), preexisting mental illness (Pan et al., 2021; Bendau et al., 2021a,b), and, to some extent, loneliness (Bu et al., 2020) pose important risk factors for mental health during the pandemic. Identified protective factors include social support, resilience, tolerance of uncertainty, and self-efficacy (Janssen et al., 2020; Matiz et al., 2020; Bendau et al., 2021b).

As evidence increases that young age is a risk factor, the present study contributes to the expanding body of research that focuses on individuals in emerging adulthood (ages 18 and older, see Arnett, 2000), a period that is already particularly vulnerable to stressors independent of societal crises such as pandemics (Arnett et al., 2014). Accordingly, in the first months of the pandemic, the largest increase in mental health problems was observed among young adults aged 18–25 years (Pierce et al., 2020; Daly et al., 2022).

In a longitudinal Irish cohort study, young adults (22 years) reported higher perceived stress and anger during the pandemic, with pre-pandemic stress being the strongest predictor for impairments and functional coping strategies (e.g., positive reappraisal, daily routine) being protective (Shanahan et al., 2022). Elmer et al. highlighted the importance of social network integration, emotional support, and interaction for undergraduate university students’ mental health (stress, depression, anxiety, loneliness), which was lower in April 2020 than in the pre-pandemic years (Elmer et al., 2020).

Likewise, a study by Preetz et al. (2021) found that emerging adults’ (21–29 years) life satisfaction and mental health were lower relative to pre-pandemic levels in a German undergraduate student sample (June/July 2020). In addition, results show heterogeneity in life satisfaction and mental health trajectories. Limited peer contacts, financial strain, and having to return to the parental home act as risk factors for longitudinal changes, and intimate partnership, social integration, and self-efficacy act as protective factors (Preetz et al., 2021).

Corroborating the age effects, young adults aged between 18 and 30 years reported a higher burden than older individuals in a longitudinal German panel study since March 2020 (Betsch et al., 2020), with a peak in January 2021, following lockdown (18–30 years:69%; >30 years: 54%). As expected, reported burden peaked again in December 2021 (18–30 years: 58%; >30 years: 49%) along with increasing COVID-19 cases and the introduction of the “3G rule” (alleviated restriction measures for vaccinated, recovered or tested individuals, only). Intriguingly, another peak in experienced burden (18–30 years: 66%; >30 years: 60%) was observed in November 2022, even though restrictions had been lifted and COVID-19 infections had declined (Betsch et al., 2020). Preliminary German data in this panel suggest that two other major crises, climate chance and the Russian war in Ukraine, were perceived as more burdensome in November 2022 (with moderate intercorrelations).^1^ In addition, non-systematic and non-peer-reviewed data from various German universities have been published online, suggesting a high burden and low overall satisfaction at different time points during the past pandemic years.^2^

## 2 Aim and research questions

The main aim of this present study was to provide further insights into the mental health, potential risk/protective factors, and pandemic-related variables among young university students during the pandemic, in order to subsequently tailor prevention and interventions more effectively to the specific challenges faced by university students. The study started in December 2020 following the first lockdown measures and increasing COVID-19 incidences. December 2022 was set as the final time point, as restriction measures had been widely abolished and the pandemic was about to be declared an endemic by decision makers in health policy. We were interested in the following research questions based on the previously cited literature:

- How do the results from December 2020 compare to pre-pandemic normative data?
- How does the time course during the pandemic unfold?
- Are there any sex differences?
- What is the impact of risk and protective factors on the severity of depression and anxiety, as examined through linear models?

We expected a higher experienced burden, along with impaired mental health and quality of life, in this emerging adult sample (Betsch et al., 2020), relative to normative data. However, it was less clear whether stricter measures and lockdowns would accompany direct increases in anxiety and depression. For example, stressful events and chronic stress have been found to predict depression, but this association varied with individual characteristics (Kessler, 1997) and might require incubation time (Bebbington et al., 1993). Therefore, the present study has a special emphasis on the risk and protective factors that have been postulated earlier (Brakemeier et al., 2020). Based on previous research findings (see Introduction), we hypothesized that females, younger individuals, and freshmen are expected to be more severely impaired. Loneliness, emotion suppression (as a dysfunctional emotion regulation strategy; Gross and John, 2003), and previous psychiatric/psychotherapeutic treatment were assumed to be risk factors, while self-esteem, resilience, and cognitive reappraisal were viewed as protective.

## 3 Methods

### 3.1 Participants and procedure

For this study, five online surveys were conducted. Students of the University of Greifswald were invited via email to participate in the first survey from December 10 to 24, 2020 (T1). An anonymous questionnaire was programmed with Evasys (Lüneburg, Germany) and participants could separately participate in a raffle (20 × 10 € vouchers). For the second survey, data were collected by Studierendenwerk Greifswald from March 1 to 19, 2021 (T2: material prizes provided by Studierendenwerk Greifswald). The students were invited via email to access the anonymous evaluation system provided by Conomic GmbH (Halle/Saale, Germany). Three more waves analogous to December 2020 were conducted between June 14 and July 5, 2021 (T3), December 1 and December 20, 2021 (T4), and December 5 and December 21, 2022 (T5). Informed consent was obtained from the participants on the first page of each survey. A total of 1.127, 760, 531, 1.226, and 814 students participated in the first, second, third, fourth, and fifth surveys, respectively (see Table 1 for description of study participants). Besides enrollment at the University of Greifswald and submission of the online questionnaire during the time periods given above, no further inclusion criteria were defined; there were no additional exclusion criteria (except for ‘other’ category in sex differences analyses, see 3.3).

**Table 1:** Sociodemographic characteristics of the samples.

	T1	T2	T3	T4	T5
*N*	1,127	760	531	1,226	814
Age group *N* (%)					
<18	1 (0.1)	0 (0)	1 (0.2)	9 (0.7)	1 (0.1)
18–22	589 (52.3)	287 (50.9)	270 (50.8)	698 (57.0)	441 (54.2)
23–27	396 (35.1)	274 (36.1)	193 (36.3)	396 (32.3)	295 (36.2)
28–32	108 (9.6)	68 (8.9)	50 (9.4)	89 (7.3)	54 (6.6)
33–37	28 (2.5)	21 (2.8)	14 (2.6)	30 (2.4)	13 (1.6)
>37	4 (0.4)	5 (0.7)	3 (0.6)	3 (0.2)	10 (1.2)
M Age	23.1 (3.9)	–^*^	23.3 (4.0)	22.6 (3.8)	22.9 (3.9)
Sex *N* (%)					
Male	290 (25.7)	193 (25.4)	128 (24.1)	275 (22.4)	200 (24.6)
Female	827 (73.4)	549 (72.2)	398 (75.0)	929 (75.8)	593 (72.9)
Other	10 (0.9)	6 (0.8)	5 (0.9)	22 (1.8)	21 (2.6)
Children *N* (%)	27 (2.4)	22 (2.9)	8 (1.5)	30 (2.4)	13 (1.6)
Desired degree					
Bachelor	424 (37.7)	280 (36.8)	207 (39.0)	420 (37.6)	324 (39.8)
Master	185 (16.4)	127 (16.7)	78 (17.7)	183 (16.3)	134 (16.4)
State examination	452 (40.1)	310 (40.8)	192 (36.2)	449 (40.1)	263 (32.3)
Other	57 (5.1)	39 (5.1)	54 (10.1)	57 (5.2)	93 (11.4)
First year	356 (32.8)	210 (28.8)	180 (34.8)	360 (30.8)	207 (25.4)
Previous psychiatric/psychotherapeutic treatment	323 (28.7)	216 (29.3)	155 (29.2)	346 (28.2)	265 (32.6)

^*^Only age groups were included in the second survey.

In 2022, a total of 10.366 students (59.2% female) were enrolled at one of five faculties (Theology: 1.3%, Law and Political Science: 20.2%, University Medicine: 22.4%, Philosophy: 23.0%, Mathematics and Natural Sciences: 33.1%). Thirty-two Bachelor’s and thirty-six Master’s degree programs as well as forty State examination programs (German Staatsexamen, e.g., teacher and law programs) were offered in 2022.

In the present study, there was a larger proportion of Natural Sciences students (Theology: 1.5%, Law and Political Science: 16.7%, University Medicine: 12.3%, Philosophy: 25.4%, Mathematics and Natural Sciences: 43.5%).^3^

### 3.2 Measures

*The mental health* questionnaires included the PHQ-9 (Kroenke et al., 2001) and GAD-7 (Toussaint et al., 2020) for depression and anxiety symptoms. *Loneliness* was measured using the UCLA Loneliness Scale (UCLA-LS) (Döring and Bortz, 1993). *Quality of Life* was assessed using the WHOQOL_BREF and WHO-5 (Gunzelmann et al., 2006; Teststatistische Prüfung und Normierung der deutschen Versionen des EUROHIS-QOL Lebensqualität-Index und des WHO-5 Wohlbefindens-Index | Diagnostica, o. J.). *Coping strategies, resilience, self-esteem, and emotion regulation* were measured using German versions of the Brief COPE (Carver, 1997) including three subscales on active-functional, cognitive-functional, and dysfunctional coping (Prinz et al., 2012), the Brief Resilience Scale (BRS; Smith et al., 2008), Rosenberg Self-Esteem-Scale (ROS-SES; von Collani and Herzberg, 2003), and Emotion Regulation Questionnaire (ERQ; Abler and Kessler, 2009).

Moreover, several pandemic-related items were asked (see Supplementary Table S1 for specific items and results) on a Likert scale ranging from 1 (not agree at all) to 5 (totally agree). Percentages reported in the results section summarize ratings 4 and 5.

The German COVID-19 Stringency Index (“[…] a composite measure based on nine response indicators including school closures, workplace closures, and travel bans, rescaled to a value from 0 to 100, 0 – lowest strictness, 100 – highest strictness”) and the number of daily new confirmed COVID-19 cases in Germany (7-day rolling average) were retrieved online on February 13 2023 from https://ourworldindata.org/ and added as variables to the dataset.

### 3.3 Analyses

Statistical analyses were performed using SPSS Version 28 (IBM; Armony, NY, United States), including descriptive statistics and two-sample *t*-tests (for comparisons with normative data, if available). First, the results from December 2020 were compared to the normative data of the questionnaires, if available.

Second, the between-subjects factor Time (T1 vs. T2 vs. T3 vs. T4 vs. T5) was introduced to assess differences between the four time points and Bonferroni-corrected *post hoc* tests were conducted. Due to the small number ‘other’ sex participants were not included in the analyses (additional between-subjects factor Sex: male vs. female was introduced to analyze sex differences).

Third, to examine the influence of the COVID-19 pandemic (Stringency Index, new cases, time), possible risk factors (sociodemographic: female, sex, young age, first-year student; loneliness, emotion suppression, previous psychiatric or psychotherapeutic treatment), and protective factors (self-esteem, cognitive reappraisal, resilience) on depressive and anxiety symptoms, two linear models were calculated to predict PHQ-9 and GAD-7 scores from the Stringency Index, new cases, time, female, age group, first-year student, UCLA-LS, ERQ subscales, ROS-SES, BRS, and previous treatment. For better comparability of the time intervals, only the December surveys (T1, T4, and T5) were included in these models.

## 4 Findings

The means and standard deviations for self-report questionnaires at all five time points are shown in Table 2.

**Table 2:** Self-report data, COVID-19 stringency index, and new COVID cases for all five time points.

	Time					
	T1	T2	T3	T4	T5	Value of *p*
PHQ-9	9.7 (5.8)	10.1 (5.8)	9.5 (5.7)	10.9 (6.0)	11.6 (6.4)	**<0.001**
GAD-7	8.3 (5.1)	7.6 (5.1)	8.4 (5.2)	9.7 (5.4)	10.4 (5.6)	**<0.001**
UCLA Loneliness Scale	2.2 (0.71)	2.3 (0.72)	2.5 (0.76)	3.1 (0.23)	2.3 (0.82)	**<0.001**
WHOQOL_BREF						
Physical health	47.1 (12.7)	44.7 (13.3)	55.7 (17.4)	68.1 (16.4)	46.8 (12.8)	**<0.001**
Mental health	58.9 (13.7)	55.5 (14.9)	58.1 (17.1)	56.8 (19.8)	56.6 (14.4)	**<0.001**
Social relationships	62.9 (22.4)	60.5 (21.8)	59.8 (22.3)	61.1 (21.5)	61.0 (23.2)	0.06
Environment	72.6 (14.3)	68.6 (14.0)	75.1 (15.5)	72.9 (14.5)	71.1 (15.3)	**<0.001**
WHO-5	55.7 (21.2)	42.4 (21.6)	53.4 (20.8)	57.1 (19.5)	59.1 (19.5)	**<0.001**
Brief COPE						
Active-functional	22.9 (5.4)	23.5 (5.3)	22.8 (5.1)	23.4 (5.2)	23.1 (5.5)	**0.029**
Cognitive-functional	17.4 (4.0)	17.1 (4.2)	16.6 (3.9)	17.3 (4.1)	17.2 (4.0)	**0.003**
Dysfunctional	10.2 (3.3)	10.5 (3.3)	10.2 (3.1)	10.7 (3.3)	10.9 (3.4)	**<0.001**
BRS	3.1 (0.84)	3.1 (0.79)	3.0 (0.84)	3.0 (0.84)	2.9 (0.89)	**<0.001**
ROS-SES	34.3 (6.7)	34.1 (7.2)	34.0 (7.3)	33.7 (7.3)	32.5 (7.3)	**<0.001**
ERQ						
Emotion suppression	3.7 (1.3)	3.6 (1.3)	3.7 (1.4)	3.8 (1.4)	3.9 (1.4)	**0.004**
Cognitive reappraisal	4.3 (1.1)	4.3 (1.1)	4.3 (1.1)	4.2 (1.1)	4.1 (1.1)	**<0.001**
Stringency Index [0–100]	73.4 (7.2)	77.8 (0)	67.6 (0)	48.0 (0.12)	14.8 (0)	**<0.001**
New COVID-19 cases^*^	269.0 (17.4)	117.3 (0)	14.5 (5.8)	662.6 (50.2)	318.4 (14.5)	**<0.001**

*M* (*SD*). ^*^Per million people. Bold highlights statistical significance.

### 4.1 Comparisons between December 2020 and normative data (pre-pandemic)

**Figure 1:**
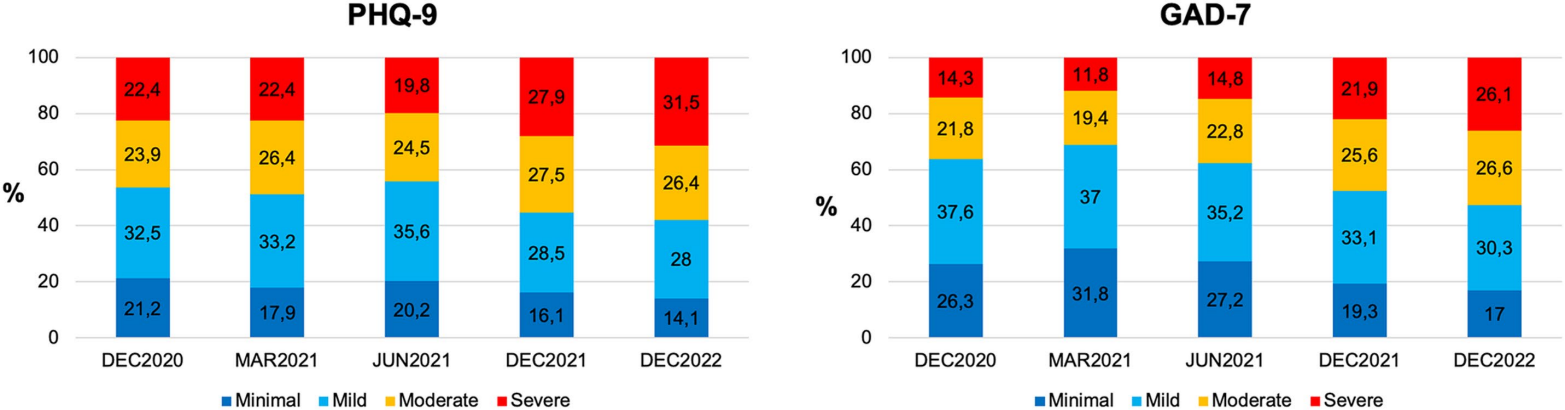
Depression and anxiety symptom severities were measured at five time points during the COVID-19 pandemic. Percentages of minimal (dark blue), mild (light blue), moderate (yellow), and severe (red) depressive symptoms (left panel) and anxiety symptoms (right panel) in December 2020; March, June, and December 2021; and December 2022.

Mean depression scores (PHQ-9) indicated mild to moderate severity (*M* = 9.7, *SD* = 5.8) in December 2020 and were significantly elevated compared to German normative data [*T* = 29.48, *df* = 1740, *p* < 0.001; (Kocalevent et al., 2013)]. 237 (21.2%) participants reported minimal, 363 (32.5%) mild, 367 (23.9%) and 250 (22.4%) severe depression (see Figure 1). Mean anxiety (GAD-7) was at the upper limit of mild severity (*M* = 8.3; *SD* = 5.1) and higher than the German normative data [*T* = 41.64, *df* = 10,836, *p* < 0.001 (Hinz et al., 2016)]. Two hundred and ninety-four participants (26.3%) reported minimal, 420 (37.6%) mild, 243 (21.8%) moderate, and 160 (14.3%) severe anxiety symptoms. In addition, loneliness (UCLA-LS; *M* = 3.1, *SD* = 0.23) was significantly higher than the German normative means (*T* = 75.33, *df* = 1707, *p* < 0.001; Döring and Bortz, 1993; *T* = 12.16, *df* = 1,421, *p* < 0.001; Klein et al., 2021).

The mean physical health (*M* = 68.6, *SD* = 6.4; *T* = −15.92, *df* = 1,355, *p* < 0.001), mental health (*M* = 59.3; *SD* = 19.5; *T* = −14.41, *df* = 1,355, *p* < 0.001), and social relationships (*M* = 62.9, *SD* = 22.4; *T* = −9.37, *df* = 1,355, *p* < 0.001) were rated lower than the normative values (WHQOL-BREF; Gunzelmann et al., 2006). However, environmental conditions were not rated lower (*M* = 72.6, *SD* = 14.3; *T* = 1.62, *df* = 1,355, *p* = 0.105).

The WHO-5 scores (*M* = 44.1, *SD* = 21.3; *T* = −32.53, *df* = 2044, *p* < 0.001) were significantly lower than the norms (Brähler et al., 2007), and the mean index value (item summary score) was 11.1 (*SD* = 5.3), indicating low well-being (<13; Brähler et al., 2007). No norms were provided for the Brief COPE scale. Resilience scores (BRS; *M* = 3.1, *SD* = 0.84) were at the lower limit to low resilience (<3; Smith et al., 2008; p. 177). Self-esteem was rated lower than in other individualistic Western countries (e.g., Pullmann and Allik; *T* = −9.80, *df* = 1,395, *p* < 0.001).

German ERQ norms were provided separately for men and women (Abler and Kessler, 2009). In December 2020, women (*M* = 3.66, *SD* = 1.4; *T* = 5.58, *df* = 1,040, *p* < 0.001) and men (*M* = 4.15, *SD* = 1.3; *T* = 3.81, *df* = 334, *p* < 0.001) showed higher emotion suppression than the German ERQ validation sample (Abler and Kessler, 2009). The use of cognitive reappraisal strategies was comparable to the norm for women (*M* = 4.30, *SD* = 1.10, *T* = 0.53, *df* = 828, *p* = 0.594) and men (*M* = 4.36, *df* = 1.16; *T* = 1.77, *df* = 0.349, *p* = 0.078).

### 4.2 Changes over time

The results for all four time points are listed in Table 2.

The percentages of depression and anxiety symptom severity are shown in Figure 1. The mean depression severity (PHQ-9) varied over time (*F*(44,366) = 16.835, *p* < 0.001, *η*^2^ = 0.015), increasing from December 2020 to December 2021 and 2022 (both *p*s < 0.001). In December 2022, depression symptom severity was higher than that in March and June 2021 (both *p*s < 0.001). The mean anxiety severity (GAD-7) also changed over time (*F*(44,373) = 37.22, *p* < 0.001, *η*^2^ = 0.033), showing an increase from December 2020 to December 2021 and 2022 (both *p*s < 0.001), a trend toward a significant increase from December 2020 to March 2021 (*p* = 0.051), and an increase between December 2021 and December 2022 (*p* = 0.036). Loneliness reports (UCLA-LS) changed over time (*F*(44,350) = 366.19, *p* < 0.001, *η*^2^ = 0.252) with maximum values in June and December 2021 (all *p*s < 0.001).

Physical health (WHOQOL_BREF; *F*(44,321) = 464.84, *p* < 0.001, *η*^2^ = 0.301) varied over time, with the highest values in June and December 2021 (all *p*s < 0.001). Relative to December 2020, mental health (*F*(44,321) = 5.77, *p* < 0.001, *η*^2^ = 0.005) was rated worse in March 2021 (*p* < 0.001), December 2021 (*p* = 0.014), and December 2022 (*p* = 0.021). In contrast, the social relationship ratings did not change over time (*F*(44,322) = 2.27, *p* = 0.06, *η*^2^ = 0.002). Environment (*F*(44,315) = 17.12, *p* < 0.001, *η*^2^ = 0.016) was rated the worst in March 2021 (all *p*s < 0.02) and the best in June 2021 (all *p*s < 0.05). The mean WHO-5 percent rank (*F*(44,374) = 80.89, *p* < 0.001, *η*^2^ = 0.069) was reduced in March 2021 relative to December 2020 (*p* < 0.001) but increased again from June 2021 (all *p*s < 0.01).

*Post-hoc* comparisons for active-functional coping in the BRIEF Cope (*F*(44,206) = 2.69, *p* = 0.029, *η*^2^ = 0.003) did not reach statistical significance. Cognitive-functional coping (*F*(44,206) = 3.97, *p* = 0.003, *η*^2^ = 0.004) was lower in June 2021 than in December 2020 (*p* = 0.003) and December 2021 (*p* = 0.005). The use of dysfunctional coping strategies (*F*(44,209) = 7.39, *p* < 0.001, *η*^2^ = 0.007) was higher in December 2022 than in December 2020 (*p* < 0.001) or June 2021 (*p* = 0.001). Resilience (BRS: *F*(44,372) = 9.26, *p* < 0.001, *η*^2^ = 0.008) was the lowest in December 2022 relative to all other time points (all *p*s < 0.01). The same pattern was observed for self-esteem (ROS-SES: *F*(44,284) = 9.00, *p* < 0.001, *η*^2^ = 0.008; *post hoc* comparisons with December 2022: all *p*s < 0.01). Emotion suppression (ERQ: *F*(44,249) = 3.86, *p* = 0.004, *η*^2^ = 0.004) was more frequently used in December 2022 than in December 2020 (*p* = 0.038) and March 2021 (*p* = 0.004). Cognitive reappraisal (*F*(44,248) = 7.353, *p* < 0.001, *η*^2^ = 0.007) was also the lowest in December 2022 relative to all other time points (all *p*s < 0.02).

**Figure 2:**
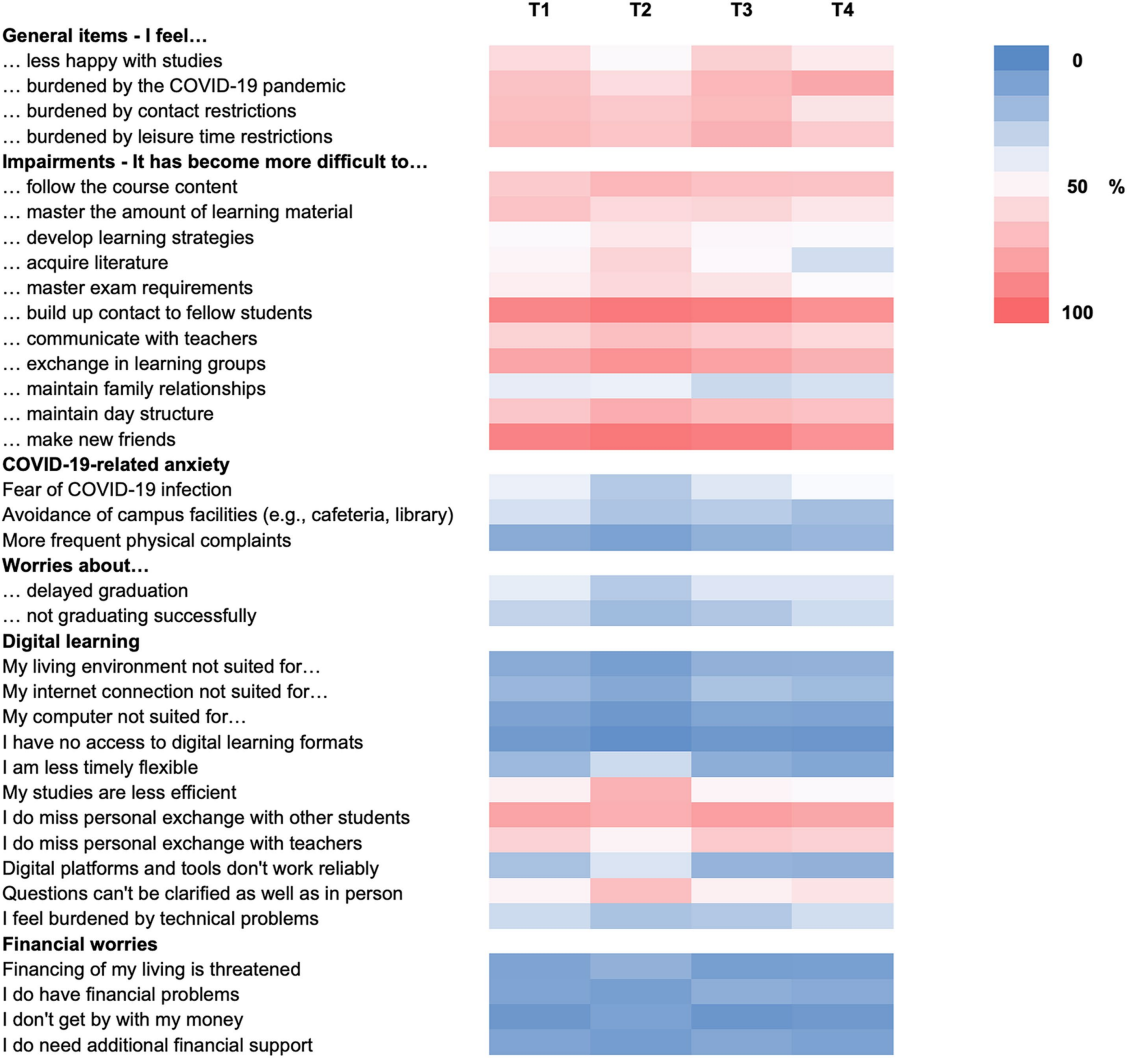
Pandemic-related items. Heat map for agreement (summarizing ratings 4 and 5) with pandemic-related items ranging from 0 (dark blue) to 100% (dark pink). Pandemic-related questions were not asked in December 2022.

Full results for pandemic-related items are presented in the Figure 2 and Supplementary Table S1. Irrespective of time, more than half of the students felt less happy and more burdened by the pandemic and its restrictions. For example, 67.8% (T1), 58.3% (T2), 71.9% (T3), and a maximum of 77.5% (T4) indicated feeling burdened by the pandemic (T5: 50.5%). Almost all students stated that building up contact to fellow students and to make new friends had become more difficult (up to 94.6%) with worst ratings in March 2021. Fear of COVID-19 infection was highest in December 2020 (41.8%) and December 2021 (45.8%), although the avoidance of campus facilities decreased (T1: 35.5%; T4: 21.4%). In December 2022, only 26.1% of students still feared COVID-19 infection. Most students (about 80%) reported having sufficient technical environment for digital learning. However, the majority missed the personal exchanges with fellow students (T1: 78.7%, T2: 74.9%, T3: 80.4, 77.7%) and teachers (T1: 61.9%, T2: 49.9%, T3: 64.8%, T4: 62.3%) during the pandemic. Approximately 10 % experienced financial worries. In December 2020, 21.7% indicated suffering from mental problems or wishes for mental treatment, and this number rose up to a maximum of 39.2% by December 2022 (T2: 24.7%; T3: 25.6%; T4: 32.3%).

Most interestingly, as pandemic burden and fear of COVID-19 infection declined in December 2022, 47.2% of students stated being burdened by Ukraine war, 65% by the climate crisis, and 79.2% by energy crisis and high inflation, indicating that other crises have come to the fore. The burden items showed significant correlations (*r* = 0.351–0.587, all *p*s < 0.001).

### 4.3 Sex differences

Women reported higher depressive (*F*(14,361) = 39.17, *p* < 0.001, *η*^2^ = 0.009) and anxiety (*F*(14,368) = 63.16, *p* < 0.001, *η*^2^ = 0.014) symptom severities than men, irrespective of time. In contrast, men reported more loneliness (*F*(14,345) = 4.425, *p* = 0.035, *η*^2^ = 0.001) in all the surveys.

Quality of life did not differ between men and women in physical and mental health subscales (all *p*s ≥ 0.263), but women were more satisfied with social relationships (*F*(14,317) = 32.02, *p* < 0.001, *η*^2^ = 0.007) and the environment (*F*(14,310) = 5.52, *p* = 0.019, *η*^2^ = 0.001). Similarly, higher WHO-5 percent ranks were observed for women (*F*(14,369) = 4.14, *p* < 0.042, *η*^2^ = 0.001), except in March 2021 (Sex × Time: *F*(44,369) = 3.25, *p* = 0.011, *η*^2^ = 0.003).

Irrespective of time, the use of active functional coping strategies was higher in women (*F*(14,201) = 57.45, *p* < 0.001; *η*^2^ = 0.013), whereas men used cognitive-functional coping more (*F*(14,201) = 13.09, *p* < 0.001, *η*^2^ = 0.003), and no sex differences were observed for dysfunctional coping strategy use (*p* = 0.724). Male participants had higher resilience (*F*(14,367) = 142.76, *p* < 0.001, *η*^2^ = 0.032) and self-esteem (*F*(14,279) = 17.87, *p* < 0.001, *η*^2^ = 0.004) ratings.

Men indicated using more emotion suppression (*F*(14,244) = 134.33, *p* < 0.001, *η*^2^ = 0.031) at all time points, but also an overall higher use of cognitive reappraisal strategies (*F*(14,243) = 3.90, *p* < 0.048, *η*^2^ = 0.001).

### 4.4 Predictors of depression and anxiety symptom severity

The regression results for the PHQ-9 and GAD-7 are reported in Tables 3, 4, respectively. Both models explained a statistically significant high proportion of the variance (PHQ-9: *R*^2^ = 0.484, *F*(123,045) = 238.15, *p* < 0.001; GAD-7: *R*^2^ = 0.421, *F*(123,045) = 184.73, *p* < 0.001). As expected, female sex, loneliness, and previous psychiatric/psychotherapeutic treatment were associated with higher depression (PHQ-9) and anxiety severity (GAD-7). In addition, first-year status was associated with higher anxiety symptoms. Higher self-esteem, resilience, and use of reappraisal strategies (at the trend level with PHQ-9: *p* = 0.065) were associated with lower depression and anxiety symptom severity. Higher number of new COVID-19 cases was associated with lower depression symptoms, whereas higher stringency index was associated with lower anxiety at trend level (*p* = 0.082).

**Table 3:** Linear model predicting PHQ-9 summary scores.

Predictor	*b*	Beta	*T*	*p*
Intercept	22.52		12.22	**<0.001**
Time	0.066	0.018	0.209	0.835
Stringency Index	−0.010	−0.037	−0.486	0.627
New COVID-19 cases	−0.003	−0.089	−2.375	**0.018**
Age	−0.035	−0.022	−1.62	0.106
Female	0.487	0.034	2.41	**0.011**
First year	0.101	0.008	0.553	0.580
Loneliness (UCLA-LS)	1.81	0.220	12.00	**<0.001**
Self-esteem (ROS-SES)	−0.341	−0.398	−22.57	**<0.001**
Cognitive reappraisal (ERQ)	−0.145	−0.027	−1.848	0.065
Emotion suppression (ERQ)	0.096	0.022	1.481	0.139
Resilience (BRS)	−1.531	−0.216	−13.22	**<0.001**
Previous psychiatric/psychotherapeutic treatment	0.770	0.058	4.19	**<0.001**

Bold highlights statistical significance.

**Table 4:** Linear model predicting GAD-7 summary scores.

Predictor	*b*	Beta	*T*	*p*
Intercept	20.83		12.02	**<0.001**
Time	−0.203	−0.063	−0.677	0.498
Stringency Index	−0.033	−0.141	−1.74	0.082
New COVID-19 cases	0.000	−0.008	−0.210	0.834
Age	−0.016	−0.011	−0.772	0.440
Female	0.481	0.038	2.67	**0.008**
First year	0.387	0.033	2.25	**0.024**
Loneliness (UCLA-LS)	1.12	0.153	7.89	**<0.001**
Self-esteem (ROS-SES)	−0.194	−0.255	−13.64	**<0.001**
Cognitive reappraisal (ERQ)	−0.187	−0.039	−2.54	**0.011**
Emotion suppression (ERQ)	−0.035	−0.009	−0.566	0.571
Resilience (BRS)	−1.18	−0.346	−20.06	**<0.001**
Previous psychiatric/psychotherapeutic treatment	0.634	0.053	3.669	**<0.001**

Bold highlights statistical significance.

## 5 Discussion

In summary, the findings indicate that in December 2020, students from Northern Germany exhibited elevated scores in depression and anxiety, along with heightened levels of loneliness and emotion suppression, compared to pre-pandemic conditions. Indicators such as self-esteem, physical and mental health, social relationships, and well-being demonstrated a decline. While no disparities emerged in pre-pandemic resilience and cognitive reappraisal strategies, it’s worth noting that resilience was positioned at the lower boundary of the normal range.

A recent meta-analysis found a significant association between social restrictions or quarantine and overall mental health impairments, depression, stress, and loneliness but not anxiety (Knox et al., 2022). However, in our own study, we did not detect comparable associations. It’s important to note that the authors of the meta-analysis highlighted concerns regarding the quality of the extensive and swift research output, as well as the contradictory nature of findings, particularly when assessing subgroups.

As expected, anxiety and depression symptom severity were elevated in our study compared with pre-pandemic levels (Elmer et al., 2020; Fancourt et al., 2021; Shanahan et al., 2022). Students reported being burdened by the disruption to educational plans and, in particular, social isolation resulting from restriction measures and lockdown. In line with this, loneliness has increased relative to pre-pandemic levels, mainly in 2021. Before (Cacioppo et al., 2006; Cacioppo and Patrick, 2008) and during the pandemic (Palgi et al., 2020; Killgore et al., 2020a), loneliness has been found to be associated with depression and anxiety. Paralleling this, self-reported loneliness predicted both PHQ-9 and GAD-7 scores in the present study. However, the extent of restriction measures (stringency index), including social distancing, did not predict the severity of depression symptoms. This supports the incubation time hypothesis (Bebbington et al., 1993), along with our finding of increasing PHQ-9 values over the December waves, while stringency index decreased. Evidence suggests that lockdowns can influence later depression, although the relationship between the two is complex and can be influenced by a range of factors. For example, loneliness and depression symptom severity remained elevated between April and June 2020, although social constraints decreased (Killgore et al., 2020b). At the trend level, a higher measure of stringency was accompanied by reduced anxiety ratings, suggesting that they were somewhat protective. Some people may experience reduced anxiety during lockdowns and social distancing measures, particularly if they have pre-existing anxiety disorders or if their anxiety is triggered by specific social or environmental stressors that are alleviated by lockdowns (Zheng et al., 2020). However, this should be interpreted with caution, in light of previous psychotherapeutic/psychiatric treatment being a major predictor of depression and anxiety symptom severity. Consistent with the present findings, many studies have found that pre-existing mental illness is a risk factor for mental health impairment during the COVID-19 pandemic (Neelam et al., 2021; Wirkner et al., 2022). For example, 60% of individuals with a history of mental illness, especially anxiety, depression, PTSD, and eating disorders, reported worsening mental health during the pandemic (Lewis et al., 2022). Unfortunately, the present study did not assess specific pre-pandemic diagnoses.

In addition to loneliness (Kohls et al., 2023) and previous treatment, we found female sex to be a risk factor for depression and anxiety symptom severity (Pierce et al., 2020; Niedzwiedz et al., 2021; Ramiz et al., 2021; Bendau et al., 2021b; for a review, see Wirkner et al., 2022). These findings are consistent with those of pre-pandemic research on sex and mental health. Women are more likely to suffer from depression and anxiety disorders than men, with the onset age peaking during adolescence and early adulthood (Altemus et al., 2014; Jacobi et al., 2014). In addition to various biological and cultural factors, experiential factors have been suggested to contribute to sex differences in anxiety and depression. Pre-clinical studies found that women tend to show higher stress reactivity, for example, are more vulnerable due to different stress neuropeptide and hormone influences (for review, see Bangasser et al., 2019), suggesting that the multidimensional stressor COVID-19 pandemic affects women differently than men.

Being a first-year student predicted higher GAD-7 scores, but not PHQ-9 scores. Freshmen may be more affected by the pandemic because the changes and disruptions experienced in their academic and social lives occur just as they are already adjusting to a new environment. For example, Spanish undergraduates reported more stress than master students during the first weeks of the pandemic, (Odriozola-González et al., 2020). Not surprisingly, age was not associated with depression or anxiety symptoms, most likely due to the homogenous group. Financial worries and technical issues during remote learning played only a minor role, as did worries about delayed graduation between December 2020 and December 2021, suggesting sufficient adaptation by responsible authorities. However, successful adaptation is highly dependent on socioeconomic and psychosocial factors, as shown in US samples (Aucejo et al., 2020; Lytle and Shin, 2023). For example, Lytle and Shin (2023) reported that grit and resilience predict fewer academic and career concerns among first-year undergraduate students during COVID-19. Thus, it is important to note that while loneliness, female sex and previous mental illness are risk factors for mental health conditions, existing protective factors might buffer the detrimental effects of (pandemic) distress on mental health. In the present study, we identified higher self-esteem and resilience as significant predictors of lower anxiety and depression severity, which is in line with the growing body of concordant literature during the pandemic (for a review, see Wirkner et al., 2022). However, this finding from the COVID-19 pandemic is not new. Resilience is the ability to flexibly adapt to stress and major life events, and is protective in early academic education (Galatzer-Levy et al., 2012), and after early (Meng et al., 2018) or later life distress (Goldmann and Galea, 2014). Moreover, there is longitudinal evidence that low self-esteem predicts depression but not anxiety, supporting the vulnerability hypothesis for depression (Orth et al., 2008; Sowislo and Orth, 2013). In response to the pandemic, promising approaches have been developed to promote resilience in young adults (Bartos et al., 2021; Rania et al., 2022).

With regard to emotion regulation, we found elevated emotion suppression, but not cognitive reappraisal, in December 2020, and declining use of functional emotion regulation strategies in December 2022. Although the heightened use of emotion suppression was not predictive of anxiety and depression symptom severity, higher cognitive reappraisal was associated with lower anxiety and depression symptom severity at the trend level. There is abundant evidence that reappraisal strategies are successful in regulating anxiety and negative valence at subjective and physiological levels (Hofmann et al., 2009; Hartley and Phelps, 2010; Hermann et al., 2014). For example, they buffer the impact of high stress on depression (Troy et al., 2010). In contrast, dysfunctional emotion regulation strategies are associated with depression and anxiety (for review, see Schäfer et al., 2017). In line with the quantitative data, qualitative research found high emotional vulnerability in young Italian university students, with more frequent reports of unpleasant emotions (including fear, anxiety, and depression; Migliorini et al., 2021). Qualitative data also revealed that individuals living in environments that were highly affected by the virus, reported more avoidance-focused coping strategies (e.g., trivialization) – in contrast to an emerging use of functional emotion- and problem-focused coping in less affected regions (Migliorini et al., 2021).

This study has some strengths, although at the same time the interpretability of the results is also affected by some limitations, which are identified below. First, the online cross-sectional design should be mentioned as a limitation of the present study. However, the study does, after all, span the past 2 pandemic years and includes five measurement time points, including December 2022 when the pandemic was about to be declared “over” and when restriction measures were relaxed. This addresses some of the recent criticisms (Knox et al., 2022). Second, participation was lower in March and June 2021, likely due to different contact formats in March 2021 and participation fatigue in surveys with higher frequency. Moreover, central mailing lists have been often used to spread information during the pandemic.^4^ For the present purpose, the online format and the use of central mailing lists had several advantages: adherence to restriction measures, low amount of missing data (all questions had to be answered before submission, except for wave two), and high reach. Third, the PHQ-9 and GAD-7 are widely applied screening tools for depression and anxiety severity. However, depression or anxiety disorder diagnoses should be used in conjunction with clinical judgment and other sources of information (Kocalevent et al., 2013). Thus, with the current data, we cannot make any statements on individual diagnoses, including further mental disorders (e.g., PTSD and eating disorders). However, literature-based mental health and related risk- and protective factors were systematically assessed, using validated instruments.^5^ Fourth, we were not able to collect all relevant variables regarding the sample: we do not have information regarding the diversity of our study sample (such as ethnicity, SES) and the history of diagnoses and treatments. However, in such surveys, the duration of completion must always be weighed against the constructs of interest in order to obtain a sufficiently large sample and data quality. Fifth and finally, we started the first survey at very short notice in the middle of the onset of the pandemic and therefore only verbally agreed on our approach with the ethics committee of the University of Greifswald. Since the study was conducted in accordance with ethical guidelines, the local legislation and institutional requirements and was only an online survey, we also waived a detailed ethics application.

## 6 Conclusion

In this study, students reported impaired mental health at 5 measurement points during the COVID-19 pandemic. Among these, students appeared to be most burdened in December 2022: 32% of participants conveyed severe depressive symptoms, and 26% conveyed severe anxiety symptoms. Key risk factors identified included female sex, loneliness, and previous mental health treatments, whereas higher self-esteem, resilience, and the utilization of cognitive emotion regulation strategies were found to be protective factors.

Even though health policymakers and other authorities, such as university chancellors and presidents, have expressed hope that mental distress would improve with the waning of the pandemic and the implementation of alleviating measures, we cannot observe this relief in our data. This can also be attributed to the persistent strains stemming from economic crises, war anxiety, and especially the climate crisis, indicating that no respite is imminent in the near future. Reducing loneliness, as well as enhancing self-esteem, resilience, and functional emotional regulation strategies, should be objectives for prevention and intervention in the realm of student mental health, with special attention to vulnerable individuals (females, pre-existing mental issues, first-year students). In this context, a multifaceted approach in higher education should encompass access to psychosocial services such as counseling and therapy, the cultivation of a sense of community through interactions (e.g., in-person classes, cafeterias, libraries, university sports, celebrations), as well as the promotion of resilience, self-care, and the application of functional emotional regulation strategies through workshops, mentorship programs, and extracurricular activities. Furthermore, financial security must always be kept in focus, and sustainability should be encouraged.

The mental health of students in the context of challenging times, particularly during the global crises, should be more intensively and systematically monitored in the future. This will allow us to continually enhance our educational and intervention offerings and respond promptly to deteriorations. Students are the architects of the future, and ensuring their psychological well-being while enhancing their self-efficacy and resilience is imperative. This endeavor not only serves their immediate well-being but also bolsters their capacity for assuming responsibility during times of crisis in the future.

## Data availability statement

The raw data supporting the conclusions of this article will be made available by the authors, without undue reservation.

## Ethics statement

Ethical approval was not required for the study involving humans in accordance with the local legislation and institutional requirements. Written informed consent to participate in this study was not required from the participants or the participants’ legal guardians/next of kin in accordance with the national legislation and the institutional requirements.

## Author contributions

JW: Conceptualization, Data curation, Formal analysis, Investigation, Methodology, Project administration, Software, Writing – original draft. E-LB: Conceptualization, Investigation, Project administration, Writing – review & editing.

## Footnotes

^1^https://projekte.uni-erfurt.de/cosmo2020/files/COSMO_W70.pdf

^2^https://www.fzs.de/2021/08/20/ueberblick-studierendenschaftsumfragen/

^3^https://www.uni-greifswald.de/en/university/information/numbers-facts/key-data-and-numbers/

^4^In June 2021, access to the central mailing list was blocked, preventing a sixth valid survey wave. Thus, in summer 2021, only 145 students participated in our survey which was advertised over social media and psychology student networks. Data are not reported here.

^5^https://www.nhlbi.nih.gov/health-topics/study-quality-assessment-tools
