# Supplementary material for: The crisis is over, long live the crisis: mental health in emerging adulthood during the course of the COVID-19 pandemic
Source: Front Psychol. 2024 Jan 31;15:1283919. doi: 10.3389/fpsyg.2024.1283919 (PMC10864646; doi:10.3389/fpsyg.2024.1283919)
Supplement: Supplementary file 1 [file Table_1.docx]

Supplementary Material

# Supplementary Table

**Supplementary Table 1.** Pandemic-related items (M, SD, %) for four time points.

|  | **T1** | | | **T2** | | | **T3** | | | **T4** | | |  |
| --- | --- | --- | --- | --- | --- | --- | --- | --- | --- | --- | --- | --- | --- |
|  | **M** | **SD** | **%** | **M** | **SD** | **%** | **M** | **SD** | **%** | **M** | **SD** | **%** | **p** |
| **General items – I feel…** |  |  |  |  |  |  |  |  |  |  |  |  |  |
| … less happy with studies | 3.6 | 1.3 | 59.1 | 3.3 | 1.4 | 48.0 | 3.7 | 1.3 | 62.9 | 3.3 | 1.7 | 53.2 | **< .001** |
| … burdened by the COVID-19 pandemic | 3.9 | 1.1 | 67.8 | 3.7 | 1.2 | 58.3 | 3.9 | 1.1 | 71.9 | 4.1 | 1.1 | 77.5 | **< .001** |
| … burdened by contact restrictions | 3.9 | 1.2 | 68.9 | 3.8 | 1.2 | 65.5 | 3.9 | 1.1 | 70.9 | 3.5 | 1.2 | 55.3 | **< .001** |
| … burdened by leisure time restrictions | 3.9 | 1.2 | 70.5 | 3.8 | 1.2 | 66.8 | 4.0 | 1.1 | 73.9 | 3.8 | 1.2 | 64.5 | **.002** |
| **Impairments – It has become more difficult to…** |  |  |  |  |  |  |  |  |  |  |  |  |  |
| … follow the course content | 3.8 | 1.1 | 64.7 | 3.9 | 1.0 | 72.0 | 3.9 | 1.2 | 68.1 | 3.9 | 1.0 | 67.3 | .226 |
| … master the amount of learning material | 3.7 | 1.1 | 37.3 | 3.8 | 1.0 | 59.4 | 3.8 | 1.1 | 61.0 | 3.7 | 1.0 | 54.8 | **.024** |
| … develop learning strategies | 3.6 | 1.1 | 47.9 | 3.7 | 1.0 | 54.3 | 3.6 | 1.1 | 49.0 | 3.5 | 1.0 | 47.7 | **.001** |
| … acquire literature | 3.7 | .96 | 49.2 | 3.8 | .92 | 61.4 | 3.6 | .97 | 48.3 | 3.3 | .92 | 34.6 | **< .001** |
| … master exam requirements | 3.4 | .92 | 51.8 | 3.8 | .92 | 59.7 | 3.7 | .94 | 55.5 | 3.5 | .93 | 47.3 | **< .001** |
| … build up contact to fellow students | 4.6 | .74 | 89.8 | 4.8 | .60 | 94.4 | 4.7 | .65 | 92.8 | 4.4 | .81 | 85.8 | **< .001** |
| … communicate with teachers | 3.8 | .99 | 62.1 | 4.0 | .91 | 69.0 | 3.9 | 1.0 | 64.6 | 3.8 | .96 | 59.6 | **< .001** |
| … exchange in learning groups | 4.3 | .90 | 78.3 | 4.5 | .82 | 85.2 | 4.3 | .90 | 49.5 | 4.2 | .94 | 74.1 | **< .001** |
| … maintain family relationships | 3.5 | .94 | 40.6 | 3.5 | .92 | 41.8 | 3.3 | .99 | 32.5 | 3.3 | 1.0 | 35.9 | **< .001** |
| … maintain day structure | 3.9 | 1.2 | 66.4 | 4.0 | 1.1 | 75.3 | 3.9 | 1.2 | 70.5 | 3.9 | 1.1 | 68.2 | **.008** |
| … make new friends | 4.7 | .70 | 90.6 | 4.8 | .56 | 94.6 | 4.7 | .84 | 92.4 | 4.4 | .84 | 84.8 | **< .001** |
| **COVID-19-related anxiety** |  |  |  |  |  |  |  |  |  |  |  |  |  |
| Fear of COVID-19 infection | 3.1 | 1.2 | 41.8 | 2.7 | 1.2 | 26.0 | 2.9 | 1.3 | 38.1 | 3.2 | 1.3 | 45.8 | **< .001** |
| Avoidance of campus facilities (e.g., cafeteria, library) | 2.7 | 1.4 | 35.5 | 2.4 | 1.4 | 23.9 | 2.4 | 1.4 | 27.2 | 2.3 | 1.3 | 21.4 | **< .001** |
| More frequent physical complaints | 2.0 | 1.1 | 13.6 | 1.8 | 1.1 | 9.8 | 2.0 | 1.2 | 16.0 | 2.1 | 1.2 | 18.4 | **< .001** |
| **Worries about…** |  |  |  |  |  |  |  |  |  |  |  |  |  |
| … delayed graduation | 2.9 | 1.5 | 40.5 | 2.4 | 1.4 | 26.2 | 2.8 | 1.5 | 37.8 | 2.8 | 1.4 | 37.7 | **< .001** |
| … not graduating successfully | 2.5 | 1.4 | 30.2 | 2.1 | 1.4 | 20.4 | 2.4 | 1.4 | 24.9 | 2.6 | 1.5 | 33.6 | **< .001** |
| **Digital learning** |  |  |  |  |  |  |  |  |  |  |  |  |  |
| My living environment not suited for… | 2.0 | 1.1 | 13.6 | 1.7 | 1.0 | 8.9 | 2.1 | 1.2 | 16.2 | 2.1 | 1.2 | 16.6 | **< .001** |
| My internet connection not suited for… | 2.2 | 1.3 | 18.2 | 1.9 | 1.2 | 12.7 | 2.3 | 1.3 | 23.3 | 2.2 | 1.3 | 20.1 | **< .001** |
| My computer not suited for… | 1.8 | 1.1 | 10.4 | 1.5 | .97 | 6.3 | 1.8 | 1.1 | 11.4 | 1.8 | 1.1 | 10.7 | **< .001** |
| I have no access to digital learning formats | 1.6 | 1.0 | 7.0 | 1.3 | .73 | 2.7 | 1.6 | .95 | 6.3 | 1.5 | .90 | 5.3 | **< .001** |
| I am less timely flexible | 2.4 | 1.2 | 19.2 | 2.7 | 1.3 | 33.3 | 2.2 | 1.1 | 14.8 | 2.2 | 1.1 | 12.0 | **< .001** |
| My studies are less efficient | 3.5 | 1.1 | 51.2 | 4.0 | 1.1 | 73.7 | 3.4 | 1.2 | 49.4 | 3.4 | 1.2 | 47.7 | **< .001** |
| I do miss personal exchange with other students | 4.2 | 1.1 | 78.7 | 4.1 | 1.1 | 74.9 | 4.2 | 1.1 | 80.4 | 4.1 | 1.1 | 77.7 | **.025** |
| I do miss personal exchange with teachers | 3.7 | 1.2 | 61.9 | 3.4 | 1.3 | 49.9 | 3.8 | 1.2 | 64.8 | 3.7 | 1.2 | 62.3 | **< .001** |
| Digital platforms and tools don't work reliably | 2.8 | 1.0 | 22.6 | 3.1 | 1.0 | 36.9 | 2.6 | 1.0 | 17.5 | 2.5 | 1.0 | 16.1 | **< .001** |
| Questions can't be clarified as well as in person | 3.4 | 1.1 | 50.0 | 3.8 | 1.1 | 68.8 | 3.4 | 1.3 | 51.3 | 3.5 | 1.2 | 55.6 | **< .001** |
| I feel burdened by technical problems | 2.8 | 1.3 | 33.3 | 2.5 | 1.2 | 23.3 | 2.8 | 1.3 | 25.7 | 2.8 | 1.3 | 34.4 | **< .001** |
| **Financial worries** |  |  |  |  |  |  |  |  |  |  |  |  |  |
| Financing of my living is threatened | 1.8 | 1.1 | 10.7 | 2.0 | 1.2 | 16.1 | 1.7 | 1.0 | 8.9 | 1.8 | 1.0 | 9.1 | **< .001** |
| I do have financial problems | 1.8 | 1.1 | 11.0 | 1.6 | 1.1 | 8.7 | 1.8 | 1.2 | 15.1 | 1.9 | 1.2 | 13.4 | **< .001** |
| I don't get by with my money | 1.7 | .92 | 5.6 | 1.9 | 1.1 | 9.8 | 1.7 | .91 | 5.1 | 1.8 | .93 | 6.4 | **< .001** |
| I do need additional financial support | 1.6 | 1.1 | 11.0 | 1.5 | 1.0 | 7.8 | 1.7 | 1.2 | 12.2 | 1.7 | 1.1 | 9.8 | **< .001** |
| I suffer from mental problems or wish for mental treatment | 2.2 | 1.3 | 21.6 | 2.4 | 1.4 | 24.7 | 2.4 | 1.4 | 25.6 | 2.6 | 1.5 | 32.0 | **< .001** |
